# The journey of numbers from attention to motor processing: an embodied account of neural response to number magnitudes

**DOI:** 10.1186/s12993-026-00361-x

**Published:** 2026-08-02

**Authors:** Saied Sabaghypour, Farhad Farkhondeh Tale Navi, Martin H. Fischer, Parvin Amini Yeganeh, Mohammad Ali Nazari, Mojtaba Soltanlou

**Affiliations:** 1 Department of Psychology, The University of Western Ontario, London, ON Canada; 2 Department of Cognitive Neuroscience, Faculty of Education and Psychology, University of Tabriz, Tabriz, Iran; 3 Division of Cognitive Sciences, Department of Psychology, University of Potsdam, Potsdam, Germany; 4 Faculty of Psychology, Shahid Beheshti University, Tehran, Iran; 5 Department of Neuroscience, Faculty of Advanced Technologies in Medicine, Iran University of Medical Sciences, Tehran, Iran; 6 Department of Psychology and Human Development, UCL Institute of Education, University College London, London, UK; 7 Centre for Educational Neuroscience, University of London, London, UK; 8 Department of Educational Psychology, Faculty of Education, University of Johannesburg, Johannesburg, South Africa

**Keywords:** Finger-counting, Attentional allocation, Motor activation, Alpha activity, ERPs

## Abstract

Embodied cognition suggests numbers are linked to physical actions and sensory experiences. Additionally, sensory processing, thoughts or actions are modulated by attentional shifts/control. This study investigates how learned motor associations in individuals (finger-counting habits) shape brain responses to digit symbols. We examined how covert attention to motor associations influences neural responses during passive viewing of symbolic numbers, using event-related potentials (ERPs) and time-frequency analysis. We examined whether the P2 component, linked to attentional processing, correlates with motor habits and alpha wave activity. Results showed stronger P2 component when number-hand associations aligned, with no significant alpha wave changes was observed. Right-starters exhibited enhanced attention and number processing, with higher P3 amplitudes and faster responses for all digits. Taken together, these findings provide preliminary support for the idea that attention to learned motor associations may influence neural responses to numerical information. We discuss these findings within our proposed theoretical framework termed Feature-based Attention to Learned Motor (FALM) processing.

## Introduction

Our inherent nature is geared towards actions. The neural mechanisms, such as selective attention, are expected to depend on our capacity for action, even if we do not have a specific action in mind. An association between attention allocation and sensory-motor activities has been studied in the embodied cognition framework [7, 87]. Earlier research has demonstrated that attention effects transfer to the motor domain [105], qualifying findings obtained with several standard attention paradigms [50].

Investigating these attentional and motor mechanisms involved in numerical processing has recently gained much interest in validating this new framework. Recent embodied theories have proposed that systematic sensory-motor activities are required for number acquisition [64, 72]. However, the exact neural mechanisms of these processes remain unknown. Examining the theoretical interpretations of attentional effects induced by symbol processing can unveil the cognitive processes involved in number representations [11].

The present study investigates the relationship between attentional allocation and learned motor processing with respect to numerical processing. We sought evidence for this link by employing a passive number viewing task in two groups of individuals with different finger counting habits. We aimed to investigate the attentional effects at the early stages of numerical processing and the relevant motor activation of cortical regions in response to number magnitudes.

In number processing research, attentional tasks have been employed to document that numbers influence early encoding-related processes. Simply presenting unpredictive small or large numbers (1 or 2 vs. 8 or 9) at a central fixation point will facilitate detecting subsequent targets in the left or right visual field, respectively [40]. This effect seemed hard to replicate [17] but a recent replication clarified that, for successful replication, numbers must be processed semantically and participants must be screened for their spatial counting preferences [99]. Importantly, those participants who count a row of objects from left to right show the typical attentional signature while those participants who count the row of objects from right to left show a significantly reversed spatial-numerical association [41, 42]. Furthermore, event-related potential (ERP) studies using attentional components (i.e., P1 and P3) extended this congruency effect between number magnitudes and lateralized target locations into the neurophysiological domain ([92, 95]. These reports suggest that the representation of numbers might influence spatial attention allocation. However, the influence of counting preferences varied unsystematically in previous works and their possible roles in such attentional allocation has not been considered [99]. If the magnitude of numbers is the factor influencing attention shift in space, then the direction of this effect might be contingent on different counting habits.

Developmental research supports the idea that numerical processing is associated with finger-counting [6], and several works have argued that abstract numerical representations are rooted in the finger-counting habits of adults [4, 32, 38, 91]. Individuals who begin counting with their right hand (referred to as right-starters) tend to link small numbers with the right side and large numbers with the left side, leading to a reduced or even reversed spatial-numerical association ([37, 38], see also [99]). Additionally, when measuring hand motor cortex activity following passive number viewing, a relationship between number magnitudes and the fingers used to enumerate them was observed [106]. Research also shows that the default finger counting routines of the majority of participants are stable over time in adults [52]. This line of research also indicates that brain regions responsible for hand movements become active during tasks such as numerical comparison [23, 82]. This embodied mapping suggests a strong link between hands and numerical representations.

Most previous studies in numerical cognition used lateralized responses, lateralized stimuli, or spatial cueing. This methodology left open the possibility that the above associative motor effects were induced by peripheral spatial processes rather than reflecting truly conceptual associations between number meaning and space [98]. However, recent behavioral work questioned the significance of active spatial responses by replicating the associative effect with central stimuli and central manual responses in a Go/No-go paradigm, thus removing spatial contamination [98], or when recording spontaneous manual grip force fluctuations instead of overt responses [71]. This novel approach clarifies that an investigation of the neurophysiological basis of attentional and motor associations in the absence of both spatial presentation and lateral motor response could provide valuable insights into the embodied nature of number processing. Regarding the sequential processing of numerical cognition (i.e., encoding, attention allocation and motor activation), it remains unknown whether attention to learned motor processes is linked to relevant cortical activations. Utilizing neuroscientific measures (such as ERPs) and task methods without motor requirements might provide more clear and uncontaminated evidence about the space-number associations at the conceptual level [112]. In the following, we shall present a summary of the research that motivated the conceptual framework and theoretical background of the current work.

### Feature-based selective attention and the P2 component

Theories of attention propose that attentional selection influences sensory competition by guiding how neural processing prioritizes stimuli relevant to our actions [29, 77, 101]. The features of stimuli might point to different dimensions, such as location or color. Importantly, these perceptual dimensions can encompass both external characteristics of stimuli and aspects of learned motor processing [30, 49]. In this regard, learning perceptual information automatically leads to an activation of the respective motor feature related to such information [49]. It is argued that motor features might be part of the stimulus representation and tied to the identity of specific objects and stimuli [14]. Hence, when attending to a spatial location or feature, neural responses to stimuli that share this specific property are enhanced [45, 94]. With respect to numerical cognition and based on embodied cognition theories, learned motor processes differ based on whether one starts to count from the left or right hand [38, 91]. In this context, it might be proposed that these learned motor processes could be viewed as features of numerical stimuli, specifically as learned motor features [51].

ERP research shows that attending to a particular stimulus requires processing multiple active features to isolate the source with the appropriate feature dimension [70, 76]. Although ERP data indicate that location-based attention occurs during the initial stages of stimuli processing (~ 100 ms after stimulus presentation, reflected in P1 and N1 components; Hillyard et al. 1995), evidence of feature-based attention has been found at a later stage (~ 150–250 ms reflected in P2 component; [70, 103]. Regarding mid-latency ERPs, the P2 component exhibits positive polarization and reaches its peak at approximately 200 milliseconds. These works revealed an enhanced posterior positivity contralateral to the side of an attentional shift [5, 62]. These lateralized components are suggested to reflect successive processing phases in the preparatory activation of relevant areas. The parietal cortex is considered a major hub for controlling attentional shifts [48, 73] and the P2 component has been reported to be closely related to exogenous attention [93]. This line of research points to the importance of the parietal P2 component in the neural mechanism underlying feature-based attention [48, 93]. Research also shows that the parietal cortex and feature-based attention are involved in guiding motor processes [36, 89].

### Motor-related asymmetry (MRA) as an index of cortical activation

The MRA index measures the difference between contra- and ipsilateral hand-related brain activity, reflecting unbalanced hemispheric oscillatory motor activations [21]. Several studies reported associations between the allocation of featural attention and hemispheric modulations of oscillatory activity within the alpha frequency range [59, 60]. It has been suggested that alpha asymmetry in the 8–12 Hz range indicates unbalanced activations in motor regions in response to imaginary hand and finger movements [83]. It is also well-known that hand and/or finger movement preparation blocks alpha rhythms, and inhibition of motor preparation synchronizes this oscillatory range [97]. Local alpha oscillations have been assumed to indicate inactivity of the cortical regions [78, 84].

Lateralized decreases in alpha power occur during the execution and imagination of unilateral motor movements. This effect demonstrates lateralization of spectral power decrease over the sensorimotor regions contralateral to the related hand [83]. This type of lateralization can be assessed by calculating the MRA, an event-related desynchronization index. It is reported that informative spatial cues might induce a relative alpha power decrease at contralateral electrodes to the expected target locations. Interestingly, this pattern reemerged at relatively later stages (300–750 ms), which might indicate ongoing attentive stimulus processing [88]. The sequential processing from feature-based attention to associated alpha lateralization might result in enhanced processing of stimuli at later stages [10].

### Perceptual processing and the P3 component

Selective discrimination of stimuli takes at a later decision stage that is reflected in the P3 component [3]. The centro-parietal P3 component occurs after target stimuli and, in general, reflects stimulus evaluation processes and resource allocation [55, 61]. Stimuli discrimination might benefit from the enhanced sequential processing and lead to larger and shorter P3 latencies in association with better detection processes [3]. Previous studies have found that attending to the same feature at two different locations leads to better stimuli detection and performance than attending to opposing features [69]. Several factors are recognized for their impact on P3 amplitude, including the significance or frequency of the stimulus, the attentional resource demands as well as processes like working memory updates and decision-making [9, 63]. P3 amplitude is larger for attended compared to unattended stimuli [28] with reported shorter latencies [58]. These results point to facilitation of stimulus perception with respect to larger amplitudes and shorter latencies of the P3 component.

### Current work

**Fig. 1:**
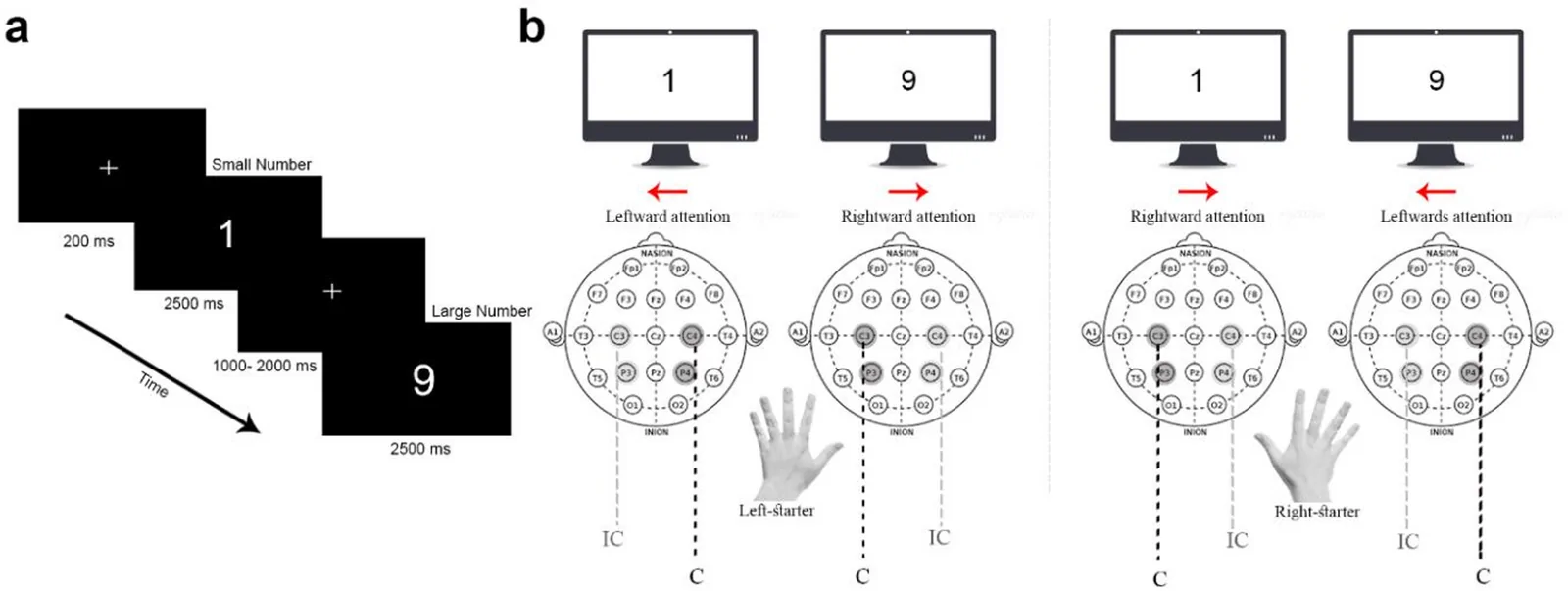
Task and study design. **a** Schematic presentation of the experiment. **b** Samples of defined congruent (*C*, dashed lines in black, dark color electrodes) and incongruent (*IC*, dashed lines in gray, light color electrodes) conditions in both left- and right-starter groups

This study investigated the influence of symbolic number presentations on attentional modulation and motor processing among participants with different finger-counting habits. In the current study, the different groups of participants [i.e., left-starters (LS) and right-starters (RS)] passively viewed small (1–4) and large (6–9) numbers (Fig. 1a) while their brain responses were recorded using EEG. We investigated the congruency relation between numbers and hands in two groups of LS and RS concerning attentional allocation and related motor processes (see Fig. 1b). The congruent conditions were defined as the following with respect to the elicitation of the P2 component in parietal electrodes (P3 and P4) and alpha suppression in sensorimotor electrodes (C3 and C4), respectively: Small numbers in the RS group (P3, C3), Large numbers in the RS group (P4, C4), Large numbers in the LS group (P3, C3), Small numbers in the LS group (P4, C4). The reverse combinations were considered incongruent conditions (see Fig. 1b; Table 1). The selection of electrodes of interest was theory-driven and defined a priori based on the study hypotheses and previous literature. Specifically, P3 and P4 were selected because attentional ERP effects associated with visuospatial orienting are predominantly observed over posterior parietal regions [48, 73]. In contrast, C3 and C4 were selected for the alpha-band analyses because they overlie the left and right sensorimotor cortices and are widely used to assess hemispheric differences in motor-cortex activation and sensorimotor alpha suppression [31, 79].

**Table 1:** Definition of congruent and incongruent conditions across groups and electrode sites

Group	Number magnitude	Congruent electrodes	Incongruent electrodes
Right-starters (rs)	Small numbers (1–4)	P3, C3	P4, C4
Right-starters (rs)	Large numbers (6–9)	P4, C4	P3, C3
Left-starters (ls)	Small numbers (1–4)	P4, C4	P3, C3
Left-starters (ls)	Large numbers (6–9)	P3, C3	P4, C4

P3/P4 electrodes were used for analyses of the P2 component, whereas C3/C4 electrodes were used for alpha-band analyses

If feature-based selection mechanisms point to the participants’ learned finger-counting habits, then we might expect the presence of alpha lateralization in motor areas. Finding alpha lateralization and its correlation with feature-based attention in congruent conditions with regard to number magnitude and space would be novel evidence that featural attention is recruited to modulate related motor regions in LS and RS individuals. For all participants we hypothesized (1) A larger P2 amplitude in the congruent compared to incongruent conditions, reflecting enhanced allocation of attention to motor features of symbolic numbers; (2) An alpha decrease in the congruent compared to incongruent conditions, reflecting elevated motor activation in, and (3) A negative correlation between the amplitude of the P2 component and alpha decrease in the congruent conditions. These three hypotheses represent the main aims of the present work. Considering the within-group differences, we expected that (4) the predicted congruency effects might be reversed in the right-starter (RS) and left-starter (LS) groups. This fourth hypothesis is exploratory due to the small sample size in each group. We were also interested in investigating whether such interactions might lead to enhanced processing of numbers at perceptual stages by examining the P3 component in the two groups.

## Materials and methods

### Participants

A total of twenty-two Farsi-speaking university students (*Mean age =* 28.7, *SD =* 3.24) participated in this experiment. Our sample size was based on previous works that investigated the reliability and stability of ERP components. Specifically, high Cronbach’s alpha values (> 0.7) were observed for different measures derived from the ERP (including the P2 and P3 components) in a sample of 19 participants after averaging 30 or more trials [104]. Moreover, it is reported that the P3 stabilizes with approximately 20 trials with 24 participants [113]. To minimize handedness-related variation, we only considered right-handed participants using the Edinburgh Handedness Inventory, with a score above 40. All participants were right-handed, with a handedness score of *M* = 94.68, *SD* = 14.15, and had normal or corrected-to-normal vision with no neurological and psychiatric disorders.

The participants were categorized into left-starters and right-starters based on their finger-counting habits [38]. They were asked to demonstrate with empty hands how they count from 1 to 10 by using their fingers. The experimenter recorded whether they started counting with their left or right hand. We did not consider the starting finger for counting (e.g., thumb or index finger). To confirm the temporal stability of finger counting habits [52], in addition to the first session (where participants were asked to count from 1 to 10 using their fingers), finger counting routines were measured using a paper-and-pencil survey. This survey, which included drawings of two palms taken from Fischer [38] for participants to mark their finger counting habits, confirmed the stability of these habits after two months. The data from three participants were excluded from the final analysis due to high levels of EEG artifacts. The data of 19 participants (10 right-starters and nine left-starters) were entered into the final statistical analysis. The local Ethical Committee (Isfahan Ethical Council, 20230324) approved the procedure which was in accordance with the Declaration of Helsinki’s ethical principles for humans. All participants signed an informed consent form and were compensated monetarily for their participation.

### Task and stimuli

Numerical stimuli consisted of small (1–4) and large (6–9) numbers that were presented for 2500 ms at the center of the screen (font: B-Titr, size = 150 pt). Before the presentation of each number, a fixation cross was shown for a random duration between 1000 and 1200 ms (Fig. 1a).

In addition to the Eastern-Arabic numbers that are used in Iran, and in order to maintain participants’ attention during the task, 10 English, as well as two Greek letters (“µ” and “π”), were present in 23.8% of trials with the same duration as the numbers (the “Alphabetics” condition). The participants were required to name only the English letters during the task and to refrain from responding to the Greek letters or numbers. Participants were tested individually while sitting approximately 70 cm from a computer screen. They were explicitly instructed that the numerical stimuli were irrelevant to the task. No feedback was given during the task. The experiment contained a total of 210 trials that were randomly presented to the participants. Each symbolic number (1–4 and 6–9) was presented 20 times, resulting in 80 trials for small numbers and 80 trials for large numbers (160 numerical trials in total; see Fig. 1a).

### EEG recording and analysis

EEG measurements were conducted using a Mitsar Company 10–20 international system. The electrode cap was equipped with 21 electrodes (Fp1, Fp2, F3, F4, C3, C4, P3, P4, F7, F8, T3, T4, T5, T6, Fz, Cz, Pz, O1, O2, and additional A1 and A2 connected to the left and right earlobes, respectively, as the reference electrodes). The EEG data was continuously recorded at a sampling rate of 500 Hz, with AFz as the ground electrode. Online filtering was applied with a band-pas of 0.3 to 40 Hz, and electrode impedances were kept below 10 KΩ. Offline data analysis was carried out using EEGLab ver. 2019 [24]. Instances of eyeblink artifacts were managed through Independent Component Analysis (ICA), where independent components related to horizontal and vertical eye movements were visually identified and removed for each participant. Trials exhibiting voltages beyond ± 80 µv at any electrode of interest were excluded from analysis, leaving 85% of the data included. Participants were included in the final analysis only if at least 50% of their total trials were artifact free, resulting in 1,326 retained trials for the Small Numbers condition (M = 69.79, SD = 12.70), 1,294 for the Large Numbers condition (M = 68.11, SD = 16.12), and 787 for the control condition combining alphabetic stimuli (M = 41.42, SD = 9.92). Continuous EEG data were segmented into epochs extending from − 200 to 2500 ms relative to stimulus onset. Baseline correction was applied using the pre-stimulus interval (− 200 to 0 ms). P2 and P3 amplitudes were quantified as mean amplitudes within predefined time windows based on the grand-average ERP waveforms and previous literature. The P2 component was measured between 100 and 250 ms at parietal electrodes (P3 and P4), whereas the P3 component was measured between 500 and 800 ms at electrode Cz. Although this P3 interval is later than that typically reported in active target-detection paradigms, the present study employed a passive-viewing design in which numerical stimuli were task-irrelevant. Previous studies have shown that late parietal positivities in passive visual-viewing paradigms can extend beyond 500 ms and may peak close to 1 s after stimulus onset [19]. Consistent with this literature, the 500–800 ms interval was selected to capture the broad positivity observed in the grand-average waveform. To evaluate cortical activation in motor regions, alpha-band activity (8–12 Hz) was analyzed at sensorimotor electrodes C3 and C4 during the 200–2000 ms post-stimulus interval. Cortical activation asymmetry was quantified using the Motor-Related Asymmetry (MRA) index, calculated as [(Mean alpha power at C3 − Mean alpha power at C4) / min(C3, C4)] × 100. Time–frequency analyses were performed in EEGLAB using ERSP analysis with Hanning-windowed Morlet wavelets. ERSPs were computed with cycles set to [3 0.8], 100 linearly spaced frequency bins between 1 and 20 Hz, and 200 output time points.

## Data analysis

To test Hypothesis 1, which predicted enhanced attentional allocation in congruent compared with incongruent conditions, mean P2 amplitudes were computed for each participant and compared using paired-samples t-tests. To test Hypothesis 2, which predicted greater motor-cortex activation in congruent compared with incongruent conditions, mean alpha-band power (8–12 Hz) was compared between congruent and incongruent conditions using paired-samples *t*-tests. To test Hypothesis 3, which predicted an association between attentional allocation and motor activation, Pearson correlation analyses were conducted between P2 amplitudes and alpha power in the congruent conditions. To evaluate Hypothesis 4 and explore potential differences between left-starters (LS) and right-starters (RS), repeated-measures analyses of variance (ANOVAs) were conducted. For the P2 analyses, numerical magnitude (small, large) and electrode (P3, P4) were included as within-subject factors, and group (RS, LS) was included as a between-subject factor. This model was chosen because the hypothesized congruency effects depended on both numerical magnitude and hemispheric electrode location. For the alpha-band analyses, a mixed ANOVA was conducted with numerical magnitude (small, large) as a within-subject factor and group (RS, LS) as a between-subject factor, using the Motor-Related Asymmetry (MRA) index as the dependent variable. For the P3 analyses, mixed ANOVAs with numerical magnitude (small, large) as a within-subject factor and group (RS, LS) as a between-subject factor were conducted separately for amplitude and latency measures.

When significant ANOVA effects were observed, Bonferroni-corrected post hoc comparisons were performed to further characterize the observed differences. In addition, planned comparisons relevant to the study hypotheses were conducted to examine differences between groups, numerical magnitudes, and electrode sites where appropriate. All statistical analyses were conducted using GraphPad Prism version 8.4.3 (GraphPad Software, Boston, MA, USA). Statistical significance was set at *p* < .05.

## Results

### Congruency effect in all participants

**Fig. 2:**
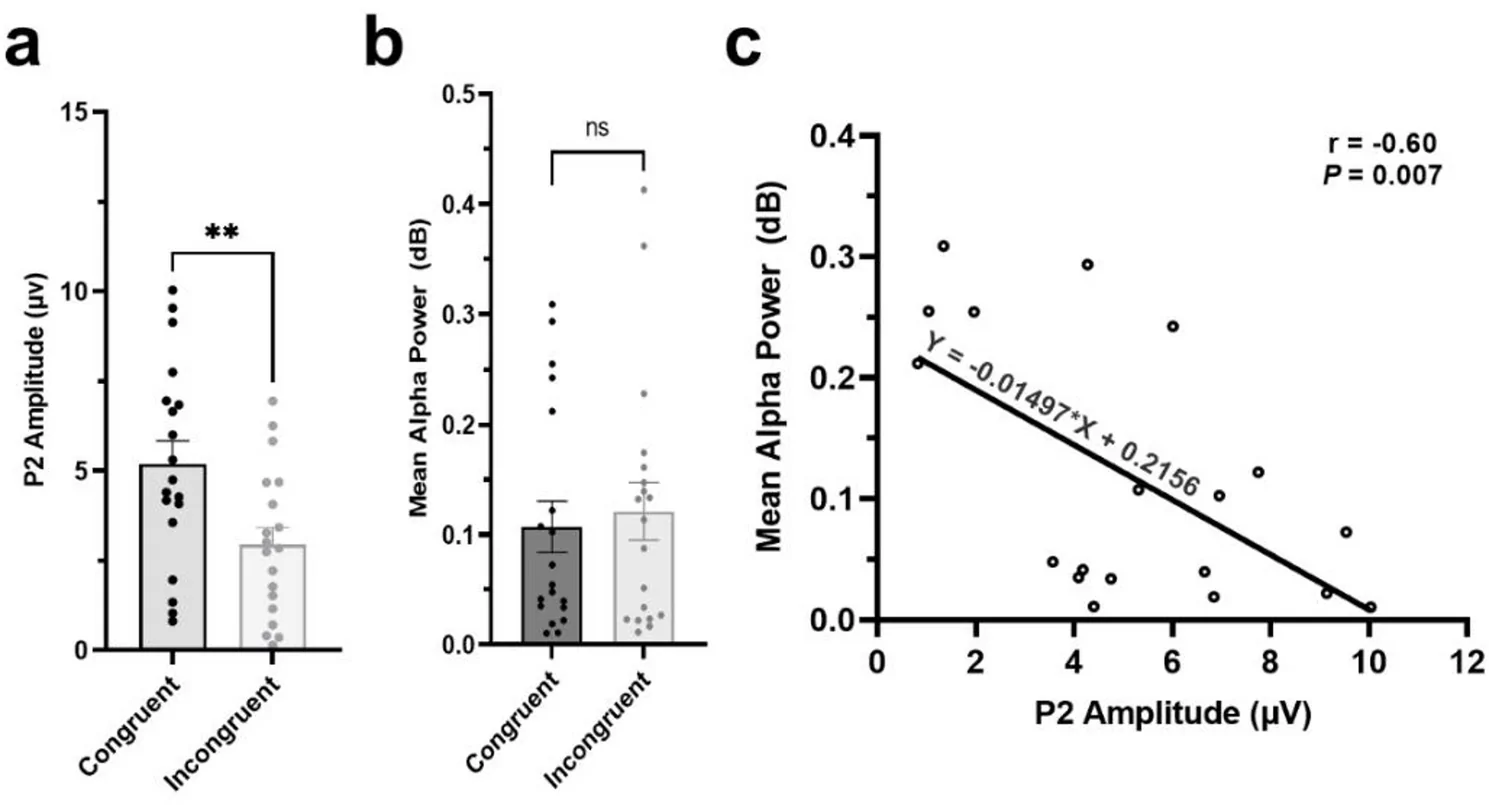
Congruency effect in all participants. **a** Differences in P2 amplitude in the congruent compared to incongruent conditions. **b** Mean alpha power differences in the congruent and incongruent conditions. **c** Pearson correlation between P2 amplitude and alpha power

With regard to our defined congruency effect and considering Hypothesis 1, we computed the means of the P2 component for each participant in congruent and incongruent conditions. Paired *t*-test revealed significant congruency among all participants for the P2 amplitude (*t(18) =* 3.42, *p* = .003, *d* = 0.91), reflecting enhanced feature-based attention at the contralateral parietal electrodes to the hands that represent numerical magnitudes in congruent conditions (*M =* 5.19, *SD =* 2.80*)* compared to incongruent conditions (*M =* 2.95, *SD =* 2.06) (Fig. 2a). The mean alpha (8–12 Hz) power was also computed for each participant for congruent and incongruent conditions. Paired *t-*test among all participants with respect to hypothesis 2, however, exhibited non-significant differences in terms of alpha mean power (*t*(18) = 1.10, *p* > .05) (Fig. 2b).

The Pearson correlation analysis between the P2 amplitude and mean power of alpha to test the hypothesis 3, indicated a significant inverse relation between enhanced feature-based attention and a decrease in alpha power in the congruent conditions (*r* = -.60, *P* = .007) (Fig. 2c). A post hoc power analysis was conducted using G*Power 3.1 (t tests, Means: Difference between two dependent means [matched pairs]; post hoc: Compute achieved power). The analysis used a two-tailed test with α = 0.05, a total sample size of 19, and an effect size of Cohen’s d_z_ = 0.78, which was calculated from the observed paired-samples t statistic (d_z_ = t/√n). The achieved statistical power was 0.9 (1 − β = 0.898; df = 18), indicating that the study was sufficiently powered to detect the observed congruency effect.

### P2 amplitude in left- and right-starters

A three-way repeated measure ANOVA considering numerical magnitude (small, large) and parietal electrode (P3, P4) as within-subject and group (RS, LS) as between-subject factors was conducted to evaluate differences in P2 amplitude for each group and condition, testing hypothesis 4. The main effect of the group was significant (*F(1*,*34)* = 8.95, *p* = .005*)*. However, there were no significant main effects of number magnitude (*F(1*,*34)* = 0.88, *p >. 05)* and electrode (*F(1*,*34)* = 2.05, *p > .05).* The interaction effect of electrode x number magnitude was found to be significant (*F(1*,*34)* = 23.41, *p <. 0001)*.

**Fig. 3:**
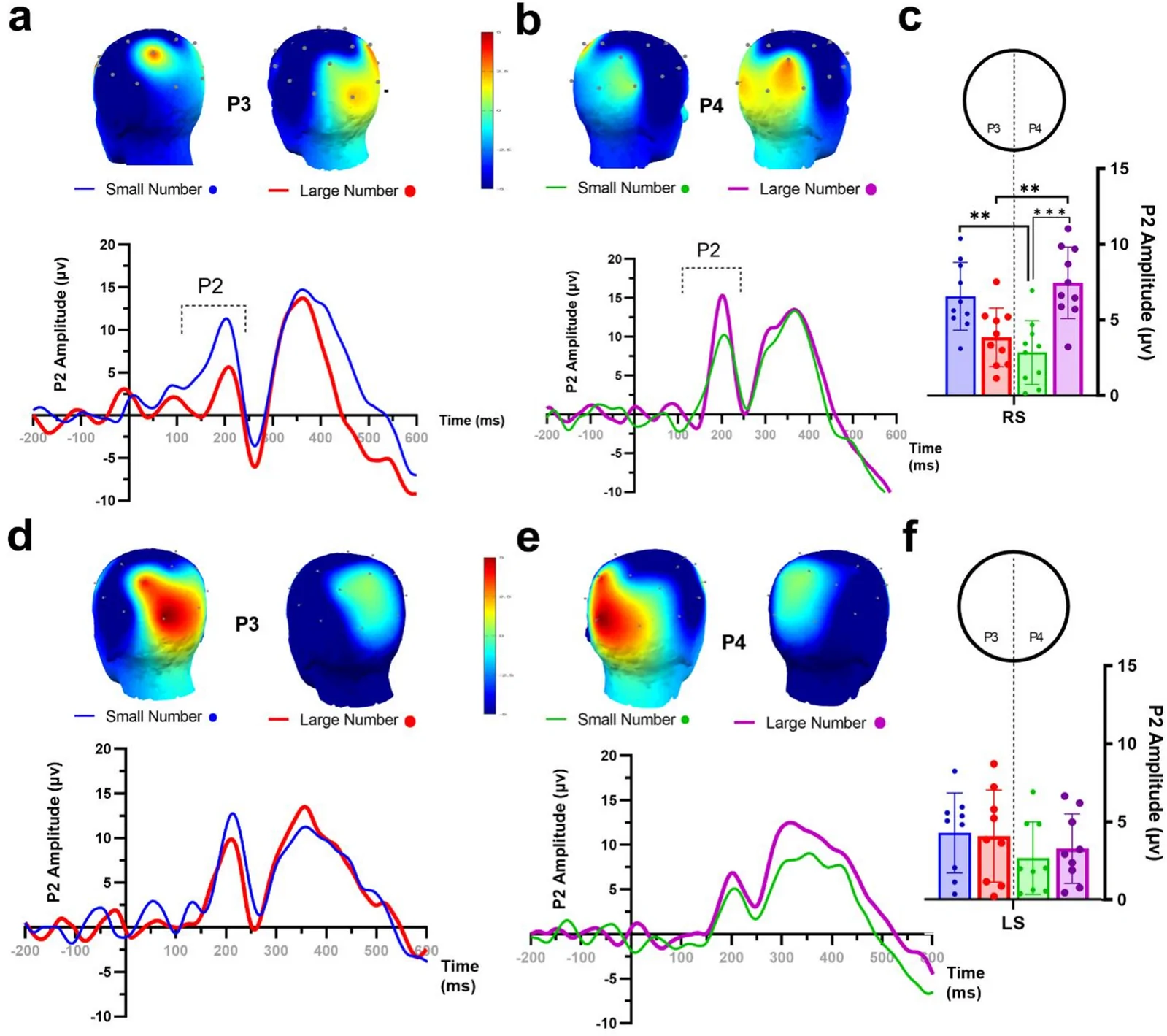
Grand average ERPs and head model maps for the RS group. **a**, **b** Comparison of small and large numbers in P3 and P4 electrodes, respectively. **c** Mean amplitude for the two channels (P3 and P4) in two conditions (Small and Large numbers). (*P<*.01: **; *P<*.001: ***). (*Mean ± SD)* (RS: right-starter; LS: left-starter)

*Post-hoc* Bonferroni’s multiple comparisons tests revealed significant differences between electrodes (P3, P4) in response to small (*t(34) =* 4.57, *p* = .0017, *d* = 1.71) and large (*t(34) =* 4.44, *p* = .0026, *d* = 1.67) numbers for the RS group. Additionally, a significant difference regarding numerical magnitude (small, large) in P4 electrode was observed (*t(68) =* 4.54, *p <* .001). Specifically, the amplitude of the P2 component was larger at the left parietal electrode (P3) in response to small numbers (*M* = 6.57, *SD =* 2.24) compared with the right parietal electrode (P4) (*M* = 2.84, *SD =* 2.11), indicating right-ward attention in response to small number (see Fig. 3a, b and c). However, a larger amplitude was observed at the right parietal (P4) in response to large numbers (*M* = 7.47, *SD =* 2.37) compared with the left parietal electrode (P3) (*M* = 3.85, *SD =* 1.93), showing left-ward attention in response to large numbers. No such effects were observed for the LS group (see Fig. 3d, e and f).

### Alpha asymmetry in left- and right-starters

To evaluate cortical activation in motor regions, alpha activity was analyzed using the sensorimotor electrodes C3 and C4. The percent of the MRA index [Mean alpha power (C3) – Mean alpha power (C4) / MIN alpha power (C3, C4) *100] was computed to evaluate the cortical activation in two groups in response to numerical magnitudes and testing hypothesis 4. A two-way repeated measure ANOVA with numerical magnitude (small, large) as within-subject and group (RS, LS) as between-subject factors was conducted. The main effect of the group was non-significant (*F(1*,*17)* = 3.35, *p* > .05). However, the main effect of numerical magnitude was significant (*F(1*,*17)* = 5.15, *p =* .036*)*.

**Fig. 4:**
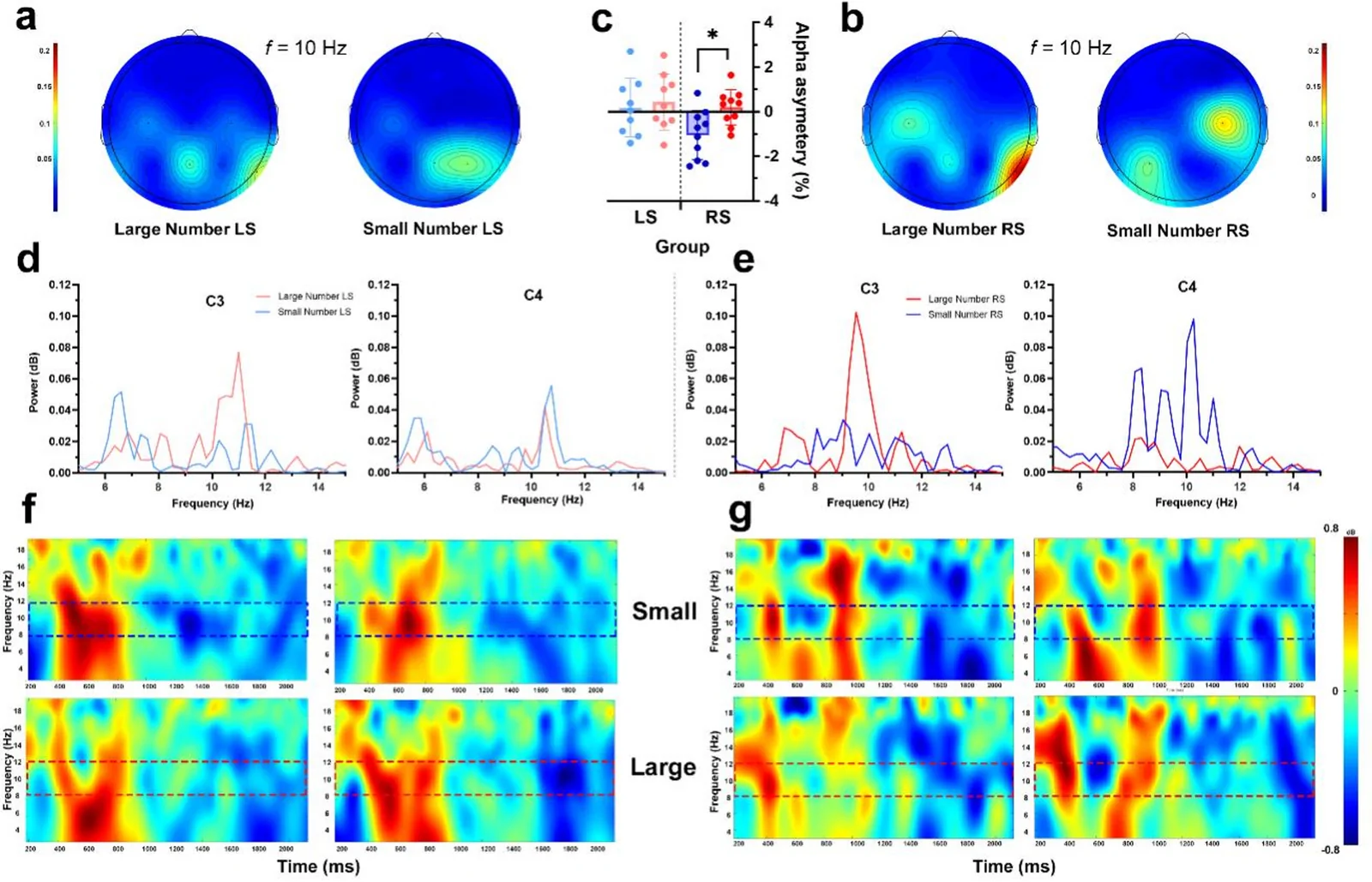
Evoked power spectrum and alpha asymmetry in response to small and large numbers in RS and LS groups. **a**, **b** Topographies at 10 Hz for the RS and LS in response to numerical magnitudes. **c** The differences in terms of the percent of the MRA index. **f**, **g** Time-frequency maps in response to small and large numbers. (RS: right-starter; LS: left-starter)

*Post-hoc* Bonferroni’s multiple comparisons test revealed a significant difference between small and large numbers in the RS group (*t(17) =* 2.74, *p* = .027, *d* = 1.30), reflecting the different effect of number magnitudes on the alpha asymmetry of motor regions. This effect was larger for small numbers. No such effect was found in the LS group (*t(17) =* 0.53, *p* > .05). The topographic maps and distribution of Alpha asymmetry in two groups are represented in Fig. 4a, b and c. The patterns of evoked alpha activity are represented in Fig. 4d and e. Figure 4f and g show the time-frequency plots for both groups (RS: left; LS: right) and numerical magnitudes (Small: top; Large: bottom).

### P3 component in left- and right-starters

To compare the RS and LS groups with respect to the perceptual stages of processing, the amplitude and latency of the P3 component were compared for the small and large numbers. To this end, a two-way repeated measure ANOVA with numerical magnitude (small, large) as a within-subject and group (RS, LS) as a between-subject factor was conducted. With regard to the P3 amplitude, the main effect of the group was significant (*F(1*,*17)* = 15.08, *p* < .01). However, the main effect of the numerical magnitude was found to be non-significant (*F(1*,*17)* = 0.36, *p* > .05). The analysis of the P3 peak latency revealed main effect of the group (*F* (1,17) = 13.21, *p* < .01). Also, the main effect of the numerical magnitude was found to be significant *(F* (1,17) = 6.20, *p* < .05).

**Fig. 5:**
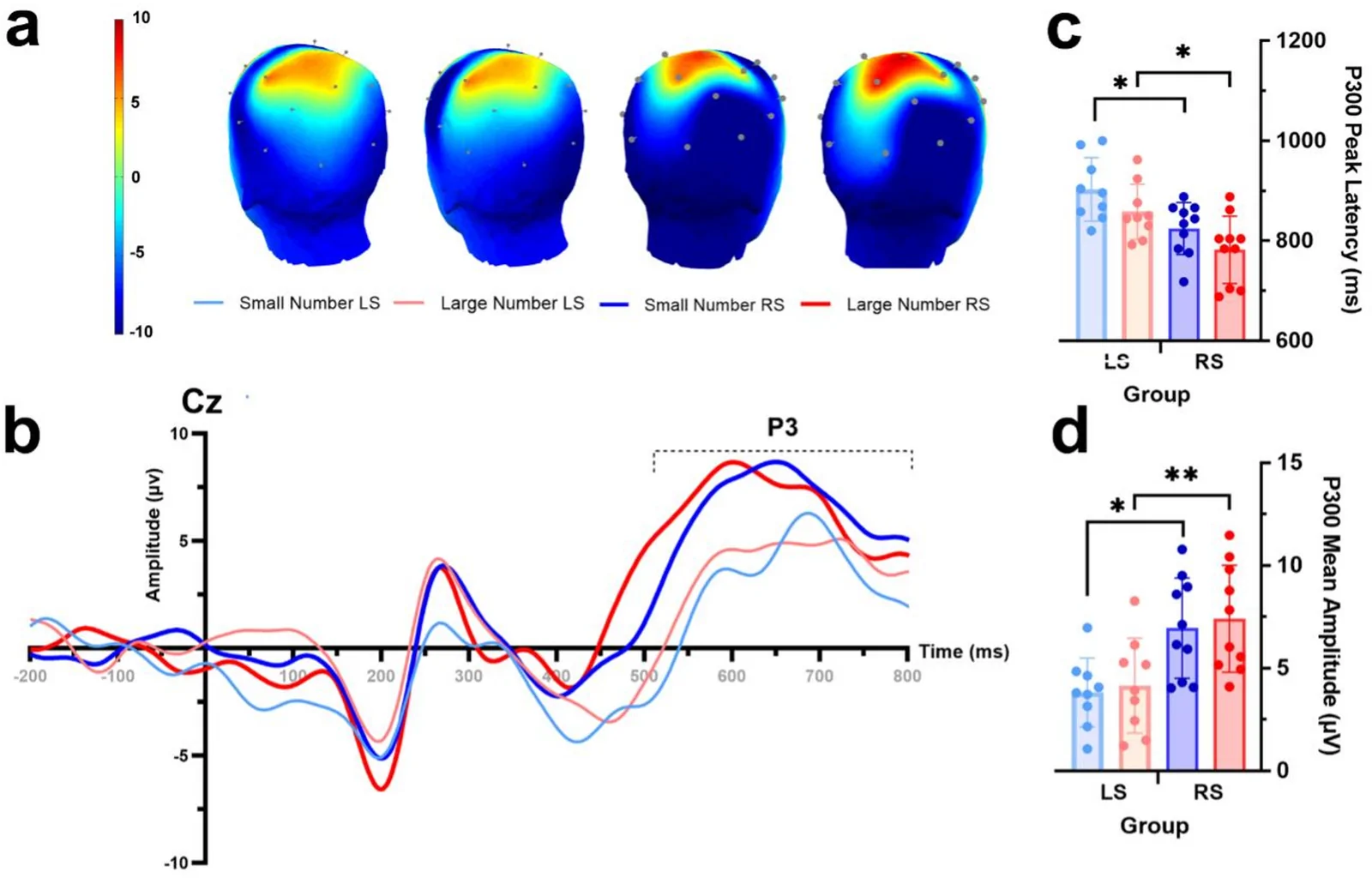
The analysis of the amplitude and the latency of P3 at the Cz electrode. **a**, **b** Head models and grand average ERPs in two groups in response to small and large numbers. **c** The differences in peak latency of the P3. **d** The differences in amplitude of the P3. (RS: right-starter; LS: left-starter)

*Post-hoc* Bonferroni’s multiple comparisons test revealed a significant difference between small numbers in the RS (*M =* 6.93, *SD =* 2.44*)* compared to the LS group (*M =* 3.82, *SD =* 1.68*)* (*t (34) =* 2.96, *p*<.05) with respect to the amplitude of the P3 component. Figure 5a and b represent the head model maps and grand average ERPs for different groups (RS and LS) and numerical magnitudes (Small and Large), respectively. A significant difference was also observed between large numbers in the RS (*M =* 7.41, *SD =* 2.60*)* compared to LS (*M =* 4.15, *SD =* 2.30*)* group (*t(34) =* 3.09, *p* < .01). In addition, there was a significant difference between small numbers in the RS (*M* = 824.60, *SD* = 52.05) compared to LS group (*M* = 902.89, *SD* = 63.53) (*t* (34) = 2.85, *p*<.05) with respect to the peak latency of the P3 component. A significant difference was also observed between large numbers in the RS (*M* = 782.20, *SD* = 67.28) compared to the LS (*M* = 858.22, *SD* = 55.23) group (*t*(34) = 2.76, *p* < .05). Figure 5c and d represent P3 latency and amplitude distribution for both groups and numerical magnitudes, respectively.

## Discussion

Our primary aim of the present work was to determine whether individual finger counting habits have an influence on covert attentional allocation and motor processing in a task with passive viewing of symbolic numbers. Two groups of left and right-starters completed the task, and the congruency effect was defined based on their finger-counting habits. The ERP analysis of the P2 component at parietal electrodes (P3, P4) indicated a congruency effect in all participants (i.e., the congruent trials elicited a larger P2 component compared to incongruent trials). The analysis of mean alpha (8–12 Hz) power at motor regions (C3, C4) did not reveal a significant congruency effect despite showing a trend in an alpha decrease in congruent trials. The correlation analysis between the P2 amplitude and mean power of alpha indicated an inverse relation that reflects a decrease in alpha power driven by feature-based attention in the congruent conditions.

Although previous works have reported that activity in the human parietal cortex is modulated by attention to different dimensions such as space and number [12, 16], they have not shown that these attentional modulations can be specific to particular features such as learned motor associations. Cell recording and neuroimaging studies have identified increased neural activity in the parietal region in both monkeys and human participants when they engage in covert attentional orienting [18, 46, 114]. Although covert-orienting of attention and preparation of motor processes such as eye movements have been reported to be dissociable from each other [54, 100], they are intricately related [47]. If attention is tightly linked to a particular motor modality, then parietal regions might also play a role in directing attention to other modalities than the eyes. This type of research has been previously referred to as motor attention to distinguish it from orienting attention [90]. Consistent with this research line, the present finding demonstrates parietal attentional modulations that are associated with numerical processing contingent on the use of specific manual motor feature information concerning finger-counting habits. Our frequency analysis in the alpha range revealed that this featural information of the numerical magnitudes may involve motor-sensitive areas [106].

Feature-based attention is important for stimuli with specific learned features. This attentional resource allocation enhances the detection and discrimination of stimuli with the attended feature [68, 110]. Previous works have shown that these attentional effects transfer to the motor domain: Attending to learned, unaware visual motion modulates motor responses compared to conditions without such attentional engagement [105]. Furthermore, the parietal areas are suggested to be involved in motor attention [89] and numerical processing [15]. The present work aimed at studying feature-based attention also implied a motor component in response to numerical presentation. Our frequency analysis in the alpha range revealed that this featural information of the numerical magnitudes may trigger motor-sensitive areas. Our within-subject analysis revealed a significant alpha asymmetry in motor areas solely in the RS group. In the context of the current experiment, where our passive stimuli contained processing of magnitudes, the presence of alpha asymmetry suggests that the same mechanism used for spatial selection is employed to amplify target processing following feature-based selection. This alpha lateralization is novel evidence that feature-based attention is recruited for the processing of numerical magnitudes. Research demonstrated that alpha activity decreases over regions on the opposite side of the target presentation versus when the target was expected ipsilaterally [59, 60]. This lateralization is thought to enhance target processing [10, 65] and reduce distractor processing [116]. Evoked alpha power analysis showed that this lateralization effect was prominent at ~ 10 Hz, corroborating recent findings regarding the association of feature-based attention and alpha activity [107]. Time-frequency analysis of the event-related data showed that the first prominent alpha desynchronization was observed ~ 600 ms after numerical presentations, which suggests that the alpha modulation in the task occurred after initial attentional selection.

With respect to our data, stimulus-relevant features can interact with each other at the perceptual level in several ways. One possibility is that attentional allocation and related motor activations are processed separately by distinct neural networks and interact through associative connections [96]. Alternatively, numerical meaning and related motor processes may be processed by a shared neural representation [66, 67].

The present findings concerning motor attention and alpha activity could suggest that habitual differences between the RS and LS groups may influence neural responses at the perceptual stage. The evaluation of the P3 in our groups indicated larger amplitudes and shorter latencies for both small and large numbers in the RS group. In this case, we speculate that the involvement of implicit attention to learned motor activities led to improved processing of numerical magnitudes in the RS compared to the LS group. However, it is important to note that these results comparing the RS and LS groups are only exploratory due to the small sample size in each group.

Several accounts have been proposed to explain the role of finger counting in numerical processing. These include their role as a memory aid during counting [102], their contribution to grasping cardinality [33], and their involvement in the development of the one-to-one correspondence principle [1]. Furthermore, it has been proposed that the visual-motor structure and movement of fingers play a part in number representations [82]. Neural specialization, which involves a stronger response to specific categories of stimuli, is established in various domains, with numbers being one such category [80]. This specialization is believed to arise from extensive experience, particularly sensory-motor experience [57] but may also reflect hemispheric predispositions [34, 35].

Our findings related to attentional modulation and consequent alpha lateralization indicated enhanced numerical processing that was reflected in both the amplitude and latency of the P3 component. This neurophysiological evidence converges nicely with behavioral studies showing that habitual finger-counting might influence how numbers are represented in the brain [26, 27]. Furthermore, variations in motor experience can result in divergent patterns of neural activation [13]. This suggests that engaging in finger counting activities plays a significant role in the brain’s specialized functions related to numbers, and having a specific finger counting practice could potentially impact this specialization.

An alternative explanation for the observed pattern regarding differences at the perceptual stage might be related to the efficacy of interhemispheric transmission, which is known to influence the amplitude and latency of the P3 component [53]. Dimond and Beaumont [25] reported that distributing perceptual load between cerebral hemispheres improved overall performance, demonstrating the benefit of interhemispheric cooperation. The primary factor influencing these advantages is related to task demands [109]. In attentionally demanding processes, between-hemispheric communications lead to faster integration of information and enhanced processing capacity [8, 81]. The observed lateralization of sequential processes in the RS group might indicate the dynamic coupling and uncoupling of numerical processing as a function of attentional demand [2, 75]. Consequently, this hemispheric advantage might have led to enhanced P3 amplitude and shorter latency in the RS compared to the LS group. Our results in this case might provide further evidence regarding a previous report on the privileges of the RS group in numerical processing [74].

**Fig. 6:**
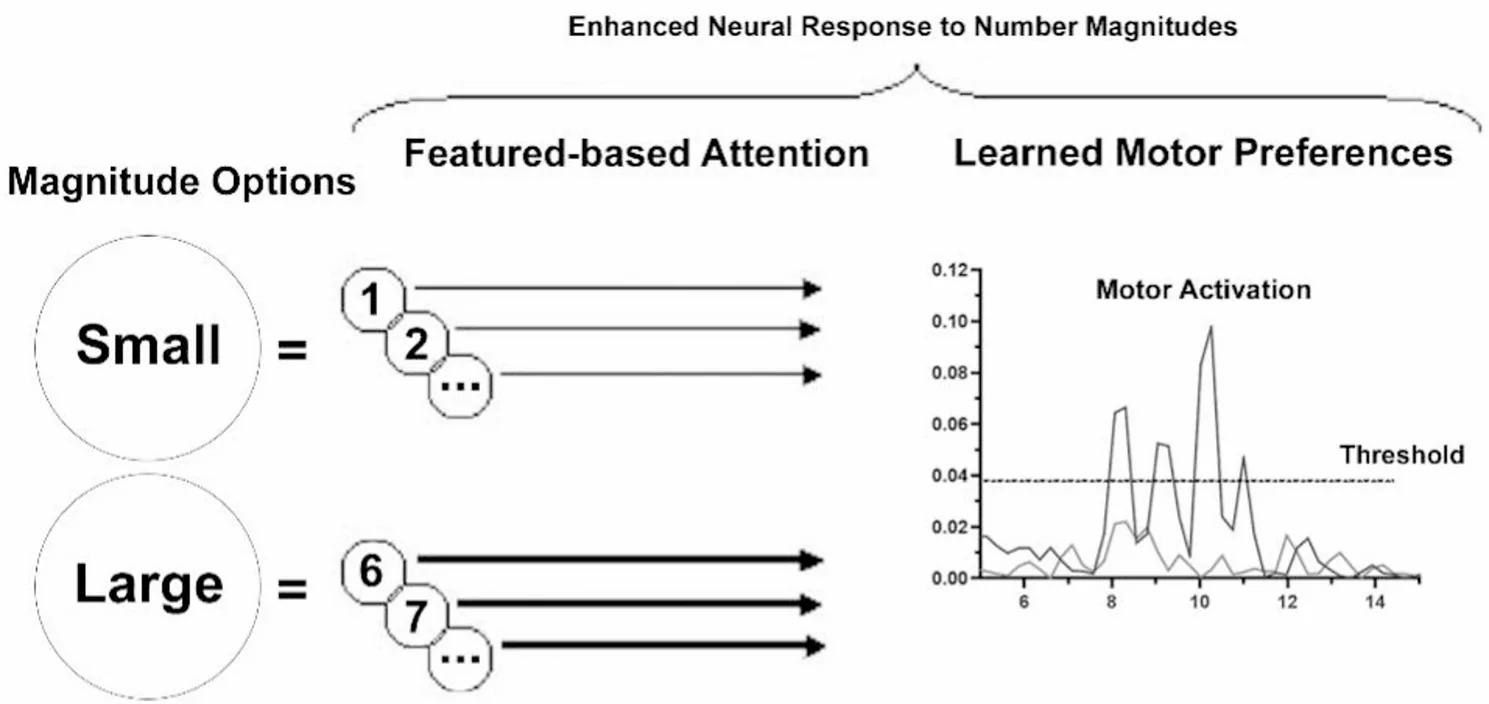
Feature-based Attention to Learned Motor Processing (FALM). The model illustrates a hypothetical mechanism by which numerical magnitudes activate learned sensory-motor associations acquired through finger-counting experience. Small and large symbolic numerical magnitudes (e.g., digits) are shown as examples of magnitude categories; in principle, the same mechanism could extend to other magnitude representations, including non-symbolic quantities. Feature-based attention is proposed to selectively enhance neural representations associated with learned motor preferences linked to a given numerical magnitude. This attentional enhancement increases activation within corresponding motor representations (right panel). The oscillatory traces represent schematic neural activity across multiple competing motor representations, whereas the dashed threshold indicates the activation level required for a motor representation to become preferentially selected. Representations receiving greater attentional enhancement are more likely to exceed this threshold and dominate subsequent processing. The figure is conceptual and is intended to illustrate the proposed interaction between numerical magnitude processing, attentional allocation, and learned motor representations

Based on the current results, it is possible that feature-based attention to learned motor processes is deployed differently in individuals with varied finger-counting habits. Theoretical and computational models of numerical processing consider the fact that numbers need to be turned into patterns of activation over a set of elementary processing units [115]. Most of the proposed models are manifestations of the number-line hypothesis, which posits that number magnitudes are encoded as points on a continuous, analogue number line [22]. In addition, in accord *with A Theory of Magnitude* (AToM) the engagement of the parietal cortex underscores the common need for spatial, temporal, and quantitative information to be employed in sensorimotor transformations [12, 43, 85, 108]. In line with this suggestion, our work might point to a theoretical framework we propose as “*Feature-based Attention to Learned Motor*” processing (FALM), which might lead to promotion of a novel model to understand the interplay between attention, motor processing, and symbolic processing (see Fig. 6). At its core, FALM posits that the way individuals deploy feature-based attention to their learned motor processes, such as finger counting habits, might play a role in shaping their neural responses to numerical magnitudes. We suggest that one specific feature of the FALM is its capacity to predict a specific time course of activations during symbolic processing. According to this framework, there is a dynamic interplay between early lateralized attentional demands and late motor activations, and this interaction may vary among individuals. FALM suggests that attentional resource allocation to learned motor activities could occur in early processing stages, influencing the preparation of relevant motor regions in a manner consistent with recent research [91] and extending earlier proposals such as *pragmatic attentional maps* or *action-centered attention* [50]. These associations might facilitate the neural response to numerical information and also lead to improved arithmetic performance [74]. This framework aligns with the broader cognitive neuroscience perspective that highlights the dynamic and interconnected nature of cognitive processes, emphasizing how learned motor associations can shape our perceptual and cognitive functions ([20, 44, 50, 56, 111]. More broadly, the proposed FALM framework is consistent with recent reviews suggesting that numerical cognition emerges through dynamic interactions between sensorimotor experience and cognitive processing across the lifespan. Recent syntheses indicate that finger-counting habits and motor experiences contribute to shaping numerical representations from childhood through older adulthood, with converging evidence pointing to number-action coupling [39, 86]. Within this broader literature, FALM provides a complementary mechanistic account by proposing that feature-based attention to learned motor representations may constitute one neural mechanism through which these long-term sensorimotor associations influence the processing of symbolic numerical magnitudes.

Several limitations of the present study should be acknowledged. First, although the number of trials was selected based on previous ERP studies, the investigation of P2 and P3 components in relation to finger-counting habits and learned sensory-motor numerical associations is still relatively novel. Therefore, future studies with larger sample sizes and a greater number of trials are needed to further establish the reliability and generalizability of the present findings. Relatedly, although previous ERP literature informed the present sample size, no formal a priori power analysis was conducted, and thus the findings should be interpreted with appropriate caution. In addition, although the proposed FALM framework offers a novel interpretation of the present findings, the framework remains theoretical and preliminary at this stage. Further replication studies using different paradigms, analytical approaches, and populations will be necessary to determine the broader applicability of the proposed model.

Overall, consistent with the general idea of embodied cognition, our results showed that finger counting might influence the way numbers are processed in individuals with different sensory-motor habits. We conclude that attention shifts in response to numerical magnitudes might be a result of previous learned motor behaviors. Also, our findings with respect to motor activations shows this attentional shift influences subsequent employment of motor regions.

## Data Availability

The data supporting the conclusions of this article can be found online at: https://osf.io/2j4az.
